# Supplementary material for: Structural and electronic properties of uranium-encapsulated Au14 cage
Source: Sci Rep. 2014 Jul 29;4:5862. doi: 10.1038/srep05862 (PMC5376176; doi:10.1038/srep05862)
Supplement: Supplementary Information [file srep05862-s1.doc]

Supporting Information

Structural and electronic properties of uranium-encapsulated Au_14_ cage

Yang Gao^1^, Xing Dai^1^, Seunggu Kang^2^, Camilo Andres Jimenez Cruz^2^, Minsi Xin^1^, Yan Meng^1^, Jie Han^1^, Zhigang Wang^1,*^ & Ruhong Zhou^1,2,3,*^

^1^Institute of Atomic and Molecular Physics, Jilin University, Changchun 130012, P. R. China.

^2^Computational Biology Center, IBM Thomas J. Watson Research Center, Yorktown Heights, NY 10598.

^3^Department of Chemistry, Columbia University, New York, NY 10027

*To whom correspondence should be addressed. E-mail: wangzg@jlu.edu.cn; ruhongz@us.ibm.com.

**Part 1.** **Two designed isomers of UAu_14_ clusters.**

**Part 2. Total bonding energy results by different functionals.**

**Part 3.** **Energy calculation for U@Au_14_ structure using spin-orbital ZORA approach.**

**Part 4. Structures of Au_2_ , U_2_, and Zr@Au_14_.**

**Part 5. The ionization potential (IP) and electron affinity (EA) of U@Au_14_ cluster.**

**Part 6. Mulliken populations of** **U@Au_14._**

**Part 7.** **The charge transfer of different systems.**

**Part 8. List of portion MOs, ordered by energy, with the most significant SFO gross populations.**

**Part 9. Occupied MOs contributed by the 5f and 6d electrons of the U atom (α orbitals only).**

**Part 1. Two designed isomers of UAu_14_ clusters.**

In addition to the U-embedded gold-cage structure U@Au_14_ in the main text, two designed isomers of UAu_14_ clusters have been considered. The first isomer, a gold-cage with a U atom adsorbed on its surface and an Au atom located inside, is marked as “iso-1”. The second isomer, an Au_14_-flake with a U atom adsorbed on its surface, is marked as “iso-2”. Similar to the structures in a previous report [29], in these two structures, the U atom prefers to move into the gold-cage, which can be clearly seen from the following Figure S1. As shown in the Table S1, the spin multiplicity of ground states of all these three structures is triplet, and the U-embedded gold-cage structure U@Au_14_ is the most stable.


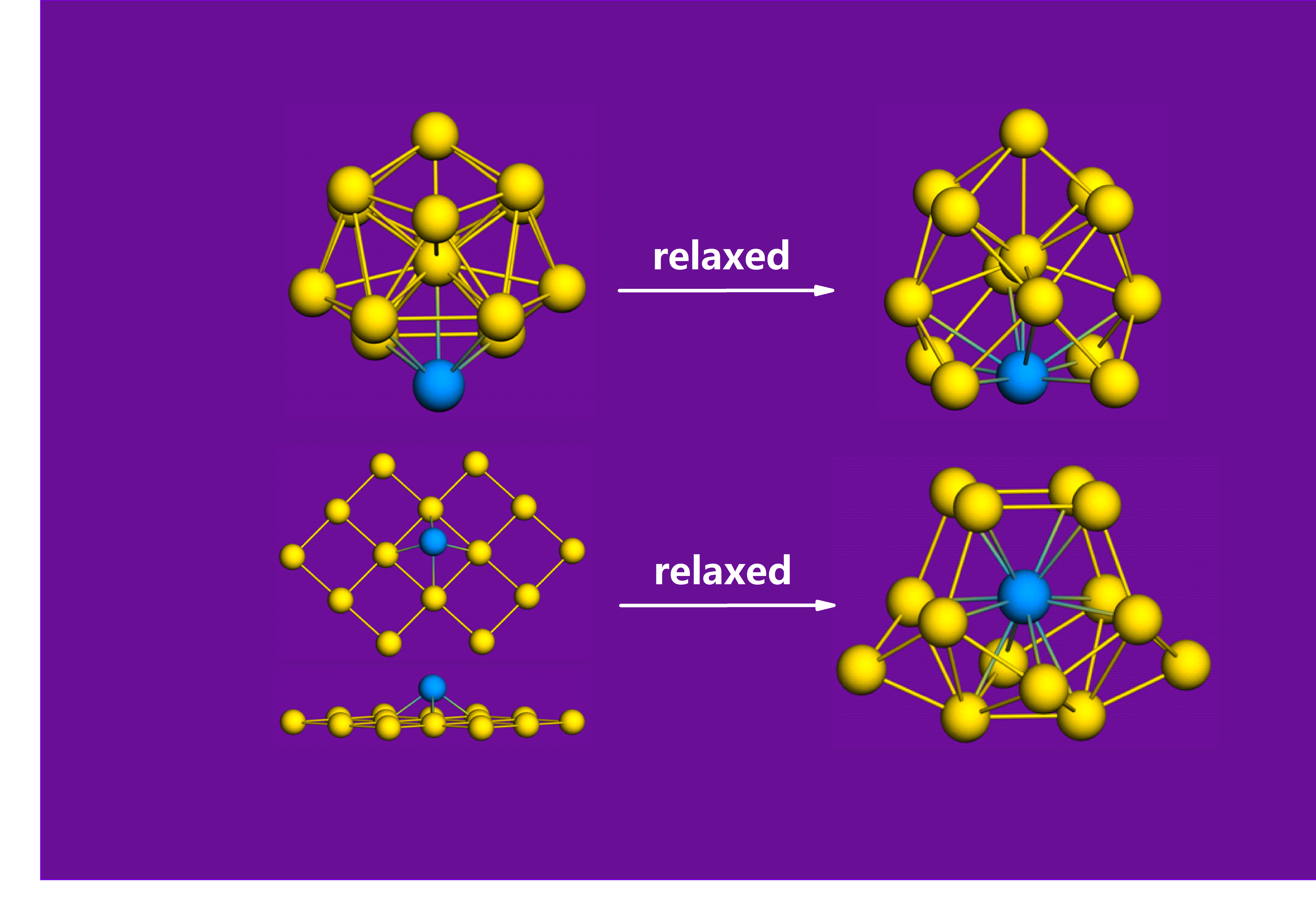


Figure S1.The relaxation process of two types of UAu_14_ structures.

Table S1. Total bonding energies results (Scalar Relativistic) of UAu_14_ isomers.

| Multiplicity | E_Iso-1_ (eV) | E_Iso-2_ (eV) | E_U@Au14_ (eV) |
| --- | --- | --- | --- |
| 1 | -40.76 | -43.08 | -44.63 |
| **3** | **-41.11** | **-43.51** | **-44.74** |
| 5 | -40.44 | -42.67 | -43.34 |
| 7 | -39.23 | -41.24 | -42.15 |

**Part 2. Total bonding energy results by different functionals.**

Table S2. Total bonding energy results by different functionals.

| System | Method | Multiplicity | Total bonding energy (eV) |
| --- | --- | --- | --- |
| U@Au_14_ | PBE | 1 | -44.63 |
|  |  | **3** | **-44.74** |
|  |  | 5 | -43.34 |
|  |  | 7 | -42.15 |
|  | BP86 | 1 | -43.67 |
|  |  | **3** | **-43.72** |
|  |  | 5 | -42.46 |
|  |  | 7 | -41.31 |
|  | PW91 | 1 | -45.13 |
|  |  | **3** | **-45.22** |
|  |  | 5 | -43.85 |
|  |  | 7 | -42.67 |
|  | B3LYP | 1 | -44.64 |
|  |  | **3** | **-45.80** |
|  |  | 5 | -43.98 |
|  |  | 7 | -42.77 |

**Part 3.**  **Energy calculation for** **U@Au_14_ structure using spin-orbital ZORA approach.**

Table S3. Relative energy calculation for U@Au_14_ using spin-orbital ZORA approach.

| System | Method | Multiplicity | △E (eV) |
| --- | --- | --- | --- |
| U@Au_14_ | PBE | 3 | 0 |
|  |  | 5 | 0.45 |
|  |  | 7 | 0.52 |

On the basis of scalar-relativistic ZORA approach optimization system, energy calculation is performed for the corresponding structures using the spin-orbital coupling ZORA approach.

**Part 4. Structures of Au_2_, U_2_, and Zr@Au_14._**

**Au_2_ and U_2._**

To check the validity of our computational method (PBE/TZ2P, scalar-relativistic ZORA), we performed the calculation on structure Au_2_ and U_2_ dimers.

For the Au_2_ dimer (Au, treating the 4f^14^5s^2^5p^6^5d^10^6s^1^ as valence electrons), the computed values of bond length, electron affinity and vibration frequency are 2.52 Å, 1.85 eV, 175.11 cm^-1^, respectively. These results are in reasonable agreement with experimental data (bond length, 2.47 Å [37]; electron affinity 1.94 eV [38]; vibration frequency, 190.09 cm^-1^ [41]), and previous theoretical results with higher-level Quantum Mechanics methods (bond length, 2.55 Å [40], 2.60 Å [39]; electron affinity 2.05 eV [39]; vibration frequency, 173.00 cm^-1^ [40]).

For the U_2_ dimer (U, treating the 5s^2^5p^6^5d^10^5f^3^6s^2^6p^6^6d^1^7s^2^ as valence electrons), the computed values of vibration frequency and bond length are 257.27 cm^-1^ and 2.28 Å, respectively. Compared with the CASSCF/CASPT2 data (265.00 cm^-1^ and 2.43 Å) from a previous report [45], our current vibration frequency is in satisfactory agreement with it. Although the bond length of U_2_ is underestimated in our DFT method, it is consistent with a previous DFT calculation (bond length, 2.27 Å [13]; 2.3-2.4 Å, [14]).

Thus, the comparison between our calculations and available past data of Au_2_ and U_2_ dimer indicates the reliability of our current approach.

**Zr@Au_14._**

A previous theoretical report indicated Zr@Au_14_ is a highly stable gold-cage metal cluster [4]. Therefore, we calculated the data of bond lengths, HOMO-LUMO gap and total bonding energy for Zr@Au_14_, at the same level (PBE/TZ2P, scalar-relativistic ZORA) with U@Au_14_. As shown in the Table S4, these results are in good agreement with the previous theoretical data. These comparisons confirmed that our current approach is reliable. Furthermore, we also compared the total bonding energy between Zr@Au_14_ and U@Au_14_, their energy values are very close, suggesting that the U@Au_14_ is a highly stable gold-cage metal cluster.

Table S4. Calculated properties of Zr@Au_14_.

| Property | Zr@Au_14_^[a]^ | Zr@Au_14_^[b]^ | U@Au_14_ |
| --- | --- | --- | --- |
| HOMO-LUMO gap (eV) | 2.23 | 2.22 | --- |
| Au-Au bond length (Å) |  |  |  |
| shortest | 2.71 | 2.74 | --- |
| longest | 2.84 | 2.85 | --- |
| Zr-Au bond length (Å) |  |  |  |
| shortest | 2.80 | 2.80 | --- |
| longest | 2.92 | 3.14 | --- |
| total bonding energy (eV) | --- | -44.15 | -44.74 |

^[a]^ Ref [4]. ^[b]^ Our calculations.

**Part 5. the ionization potential (IP) and electron affinity (EA) of U@Au_14_ cluster.**

E_VIP_, E_AIP_, E_VEA_ and E_AEA_ are defined as following:

E_VIP_ = E^+^(sp) – E^0^(opt) E_AIP_ = E^+^(opt) – E^0^(opt)

E_VEA_ = E^0^(opt) – E^-^(sp)  E_AEA_ = E^0^(opt) – E^-^(opt)

where E^0^(opt) is total bonding energy of the neutral U@Au_14_ cluster, while E^+^(opt) and E^-^(opt) represent total bonding energy of the cation and anion in optimized neutral geometry, respectively; E^+^(sp) and E^-^(sp) represent total bonding energy of the cation and anion in single point neutral geometry, respectively.

Table S5. Calculated ionization potentials (eV) and electron affinities (eV) of U@Au_14_ cluster.

| Method | Structure | E_VIP_ | E_AIP_ | E_VEA_ | E_AEA_ |
| --- | --- | --- | --- | --- | --- |
| PBE | U@Au_14_ | 6.79 | 6.78 | 2.68 | 2.73 |
| experimental value [Ref 5] | W@Au_12_ | --- | --- | 2.08±0.02 | |

**Part 6.** **Mulliken populations of U@Au_14_.**

Table S6. Mulliken populations of U@Au_14_.

| Number/Atom | Charge (e) | Spin density | Spin  direction | s | p | d | f |
| --- | --- | --- | --- | --- | --- | --- | --- |
| 1 Au | 0.12 | 0.01 | α | 1.45 | 3.19 | 4.79 | 7.01 |
|  |  |  | β | 1.45 | 3.18 | 4.79 | 7.01 |
| 2 Au | 0.12 | -0.00 | α | 1.46 | 3.18 | 4.79 | 7.01 |
|  |  |  | β | 1.46 | 3.17 | 4.79 | 7.01 |
| 3 Au | 0.13 | -0.01 | α | 1.45 | 3.18 | 4.78 | 7.01 |
|  |  |  | β | 1.46 | 3.18 | 4.79 | 7.01 |
| 4 Au | 0.12 | -0.00 | α | 1.46 | 3.18 | 4.79 | 7.01 |
|  |  |  | β | 1.46 | 3.17 | 4.79 | 7.01 |
| 5 Au | 0.13 | -0.00 | α | 1.45 | 3.19 | 4.79 | 7.01 |
|  |  |  | β | 1.45 | 3.19 | 4.79 | 7.01 |
| 6 Au | 0.13 | 0.01 | α | 1.45 | 3.18 | 4.79 | 7.01 |
|  |  |  | β | 1.45 | 3.18 | 4.79 | 7.01 |
| 7 Au | 0.12 | -0.01 | α | 1.46 | 3.18 | 4.78 | 7.01 |
|  |  |  | β | 1.46 | 3.18 | 4.79 | 7.01 |
| 8 Au | 0.11 | -0.01 | α | 1.45 | 3.20 | 4.78 | 7.01 |
|  |  |  | β | 1.45 | 3.20 | 4.79 | 7.01 |
| 9 U | -1.68 | 2.03 | α | 2.27 | 6.24 | 6.90 | 2.45 |
|  |  |  | β | 2.26 | 6.23 | 6.81 | 0.52 |
| 10 Au | 0.14 | -0.01 | α | 1.46 | 3.17 | 4.78 | 7.01 |
|  |  |  | β | 1.46 | 3.17 | 4.79 | 7.01 |
| 11 Au | 0.12 | -0.00 | α | 1.45 | 3.18 | 4.80 | 7.01 |
|  |  |  | β | 1.45 | 3.18 | 4.80 | 7.01 |
| 12 Au | 0.14 | -0.01 | α | 1.46 | 3.17 | 4.78 | 7.01 |
|  |  |  | β | 1.46 | 3.17 | 4.79 | 7.01 |
| 13 Au | 0.08 | -0.01 | α | 1.46 | 3.18 | 4.79 | 7.01 |
|  |  |  | β | 1.47 | 3.18 | 4.80 | 7.01 |
| 14 Au | 0.12 | 0.01 | α | 1.45 | 3.18 | 4.81 | 7.01 |
|  |  |  | β | 1.45 | 3.17 | 4.80 | 7.01 |
| 15 Au | 0.10 | -0.00 | α | 1.46 | 3.18 | 4.80 | 7.01 |
|  |  |  | β | 1.46 | 3.18 | 4.80 | 7.01 |

**Part 7.** **The charge transfer of different systems.**

Table S7. The charge transfer of different systems.

| **U@Au_4_** | |
| --- | --- |
| Number/Atom | Charge (e) |
| 1 Au | -0.22 |
| 2 Au | -0.21 |
| 3 Au | -0.22 |
| 4 Au | -0.22 |
| **5 U** | **0.87** |
| **U@Au_6_** | |
| Number/Atom | Charge (e) |
| 1 Au | -0.05 |
| 2 Au | -0.17 |
| 3 Au | -0.19 |
| 4 Au | -0.04 |
| 5 Au | -0.17 |
| 6 Au | -0.04 |
| **7 U** | **0.66** |
| **U@Au_8_** | |
| Number/Atom | Charge (e) |
| 1 Au | 0.01 |
| 2 Au | 0.01 |
| 3 Au | 0.01 |
| 4 Au | 0.01 |
| 5 Au | 0.01 |
| 6 Au | 0.01 |
| 7 Au | 0.01 |
| 8 Au | 0.01 |
| **9 U** | **-0.08** |
| **U@Au_12_** | |
| Number/Atom | Charge (e) |
| 1 Au | 0.10 |
| 2 Au | 0.11 |
| 3 Au | 0.11 |
| 4 Au | 0.10 |
| 5 Au | 0.10 |
| 6 Au | 0.11 |
| 7 Au | 0.10 |
| 8 Au | 0.10 |
| 9 Au | 0.10 |
| 10 Au | 0.10 |
| 11 Au | 0.10 |
| 12 Au | 0.10 |
| **13 U** | **-1.23** |
| **U@Au_16_** | |
| 1 Au | 0.17 |
| 2 Au | 0.17 |
| 3 Au | 0.09 |
| 4 Au | 0.17 |
| 5 Au | 0.09 |
| 6 Au | 0.09 |
| 7 Au | 0.09 |
| 8 Au | 0.09 |
| 9 Au | 0.09 |
| 10 Au | 0.09 |
| 11 Au | 0.09 |
| 12 Au | 0.15 |
| 13 Au | 0.09 |
| 14 Au | 0.09 |
| 15 Au | 0.09 |
| 16 Au | 0.09 |
| **17 U** | **-1.74** |

All structures are relaxed to the status of lowest energy in PBE/TZ2P level. All obtained structures were then analyzed with vibration frequency calculations at the same level to avoid imaginary frequencies and ensure the reliability of the results. For the M@Au_n_ structure, Ref S1 pointed out that it was more stable when the number of gold atoms n is even. Therefore, n was given the values of 4, 6, 8, 12 and 16 for comparison. Calculation shows that with the increment of n, the direction of charge transfer changes, and this pattern is consistent with those found in previous studies [44,S2].

**Part 8. List of portion MOs, ordered by energy, with the most significant SFO gross populations.**

**Table S8. List of portion MOs, ordered by energy, with the most significant SFO gross populations.**

| E (eV) | MO | Percentages (%) | SFO | Fragment |
| --- | --- | --- | --- | --- |
| -5.63 | HOMOα-1 | 9.87% | **6d** | **U** |
|  |  | 32.76% | 6s | Au_14_ |
|  |  | 17.17% | 5d | Au_14_ |
| -5.67 | HOMOα-2 | 10.64% | **6d** | **U** |
|  |  | 30.15% | 6s | Au_14_ |
|  |  | 16.70% | 5d | Au_14_ |
| -5.70 | HOMOα-3 | 9.36% | **6d** | **U** |
|  |  | 32.42% | 6s | Au_14_ |
|  |  | 12.52% | 5d | Au_14_ |
| -5.73 | HOMOα-4 | 10.52% | **6d** | **U** |
|  |  | 32.86% | 6s | Au_14_ |
|  |  | 10.41% | 5d | Au_14_ |
| -5.83 | HOMOα-5 | 9.24% | **6d** | **U** |
|  |  | 28.71% | 6s | Au_14_ |
|  |  | 18.96% | 5d | Au_14_ |
| -7.36 | HOMOα-30 | 1.18% | **5f** | **U** |
|  |  | 85.48% | 5d | Au_14_ |
| -7.60 | HOMOα-34 | 1.23% | **5f** | **U** |
|  |  | 81.76% | 5d | Au_14_ |
| -7.63 | HOMOα-35 | 3.00% | **5f** | **U** |
|  |  | 79.36% | 5d | Au_14_ |
| -7.68 | HOMOα-37 | 1.69% | **5f** | **U** |
|  |  | 81.30% | 5d | Au_14_ |
| -7.89 | HOMOα-38 | 7.62% | **5f** | **U** |
|  |  | 77.95% | 5d | Au_14_ |
| -7.98 | HOMOα-39 | 3.33% | **5f** | **U** |
|  |  | 81.18% | 5d | Au_14_ |
| -8.09 | HOMOα-41 | 1.36% | **5f** | **U** |
|  |  | 79.79% | 5d | Au_14_ |
| -8.19 | HOMOα-45 | 1.49% | **5f** | **U** |
|  |  | 80.45% | 5d | Au_14_ |
| -9.06 | HOMOα-58 | 1.08% | **5f** | **U** |
|  |  | 81.76% | 5d | Au_14_ |
| -9.40 | HOMOα-66 | 1.44% | **5f** | **U** |
|  |  | 6.86% | 6s | Au_14_ |
|  |  | 69.18% | 5d | Au_14_ |
| -9.50 | HOMOα-67 | 1.18% | **6d** | **U** |
|  |  | 1.24% | **5f** | **U** |
|  |  | 7.35% | 6s | Au_14_ |
|  |  | 68.24% | 5d | Au_14_ |
| -9.52 | HOMOα-68 | 1.49% | **6d** | **U** |
|  |  | 1.21% | **5f** | **U** |
|  |  | 6.17% | 6s | Au_14_ |
|  |  | 74.37% | 5d | Au_14_ |
| -9.53 | HOMOα-69 | 1.05% | **5f** | **U** |
|  |  | 1.19% | 6s | Au_14_ |
|  |  | 77.12% | 5d | Au_14_ |
| -9.97 | HOMOα-74 | 15.91% | **6d** | **U** |
|  |  | 7.21% | 6s | Au_14_ |
|  |  | 56.87% | 5d | Au_14_ |
| -10.05 | HOMOα-75 | 17.14% | **6d** | **U** |
|  |  | 9.54% | 6s | Au_14_ |
|  |  | 53.01% | 5d | Au_14_ |
| -10.13 | HOMOα-76 | 15.66% | **6d** | **U** |
|  |  | 10.89% | 6s | Au_14_ |
|  |  | 58.06% | 5d | Au_14_ |
| -10.14 | HOMOα-77 | 14.39% | **6d** | **U** |
|  |  | 11.04% | 6s | Au_14_ |
|  |  | 52.04% | 5d | Au_14_ |
| -10.16 | HOMOα-78 | 15.80% | **6d** | **U** |
|  |  | 12.32% | 6s | Au_14_ |
|  |  | 47.32% | 5d | Au_14_ |
|  |  |  |  |  |
| -5.61 | HOMOβ-1 | 9.17% | **6d** | **U** |
|  |  | 33.13% | 6s | Au_14_ |
|  |  | 16.69% | 5d | Au_14_ |
| -5.65 | HOMOβ-2 | 9.26% | **6d** | **U** |
|  |  | 30.70% | 6s | Au_14_ |
|  |  | 15.64% | 5d | Au_14_ |
| -5.68 | HOMOβ-3 | 8.91% | **6d** | **U** |
|  |  | 32.39% | 6s | Au_14_ |
|  |  | 14.27% | 5d | Au_14_ |
| -5.71 | HOMOβ-4 | 9.85% | **6d** | **U** |
|  |  | 32.87% | 6s | Au_14_ |
|  |  | 11.45% | 5d | Au_14_ |
| -5.82 | HOMOβ-5 | 8.73% | **6d** | **U** |
|  |  | 29.52% | 6s | Au_14_ |
|  |  | 18.56% | 5d | Au_14_ |
| -7.60 | HOMOβ-34 | 1.10% | **5f** | **U** |
|  |  | 82.71% | 5d | Au_14_ |
| -7.62 | HOMOβ-35 | 1.19% | **5f** | **U** |
|  |  | 81.22% | 5d | Au_14_ |
| -7.66 | HOMOβ-37 | 1.31% | **5f** | **U** |
|  |  | 80.77% | 5d | Au_14_ |
| -7.85 | HOMOβ-38 | 5.26% | **5f** | **U** |
|  |  | 81.46% | 5d | Au_14_ |
| -7.96 | HOMOβ-39 | 2.69% | **5f** | **U** |
|  |  | 80.96% | 5d | Au_14_ |
| -9.36 | HOMOβ-64 | 1.04% | **5f** | **U** |
|  |  | 2.33% | 6s | Au_14_ |
|  |  | 73.55% | 5d | Au_14_ |
| -9.47 | HOMOβ-67 | 1.45% | **6d** | **U** |
|  |  | 7.19% | 6s | Au_14_ |
|  |  | 73.71% | 5d | Au_14_ |
| -9.52 | HOMOβ-69 | 1.11% | **6d** | **U** |
|  |  | 1.60% | 6s | Au_14_ |
|  |  | 77.20% | 5d | Au_14_ |
| -9.34 | HOMOβ-74 | 14.67% | **6d** | **U** |
|  |  | 8.59% | 6s | Au_14_ |
|  |  | 56.48% | 5d | Au_14_ |
| -10.00 | HOMOβ-75 | 14.84% | **6d** | **U** |
|  |  | 8.30% | 6s | Au_14_ |
|  |  | 56.18% | 5d | Au_14_ |
| -10.07 | HOMOβ-76 | 13.47% | **6d** | **U** |
|  |  | 12.01% | 6s | Au_14_ |
|  |  | 59.11% | 5d | Au_14_ |
| -10.11 | HOMOβ-77 | 13.88% | **6d** | **U** |
|  |  | 10.12% | 6s | Au_14_ |
|  |  | 51.13% | 5d | Au_14_ |
| -10.13 | HOMOβ-78 | 14.42% | **6d** | **U** |
|  |  | 11.11% | 6s | Au_14_ |
|  |  | 49.99% | 5d | Au_14_ |

**Part 9. Occupied MOs contributed by the 5f and 6d electrons of the U atom (α orbitals only)**

Table S9. Occupied MOs contributed by the 5f and 6d electrons of the U atom (α orbitals only).

| **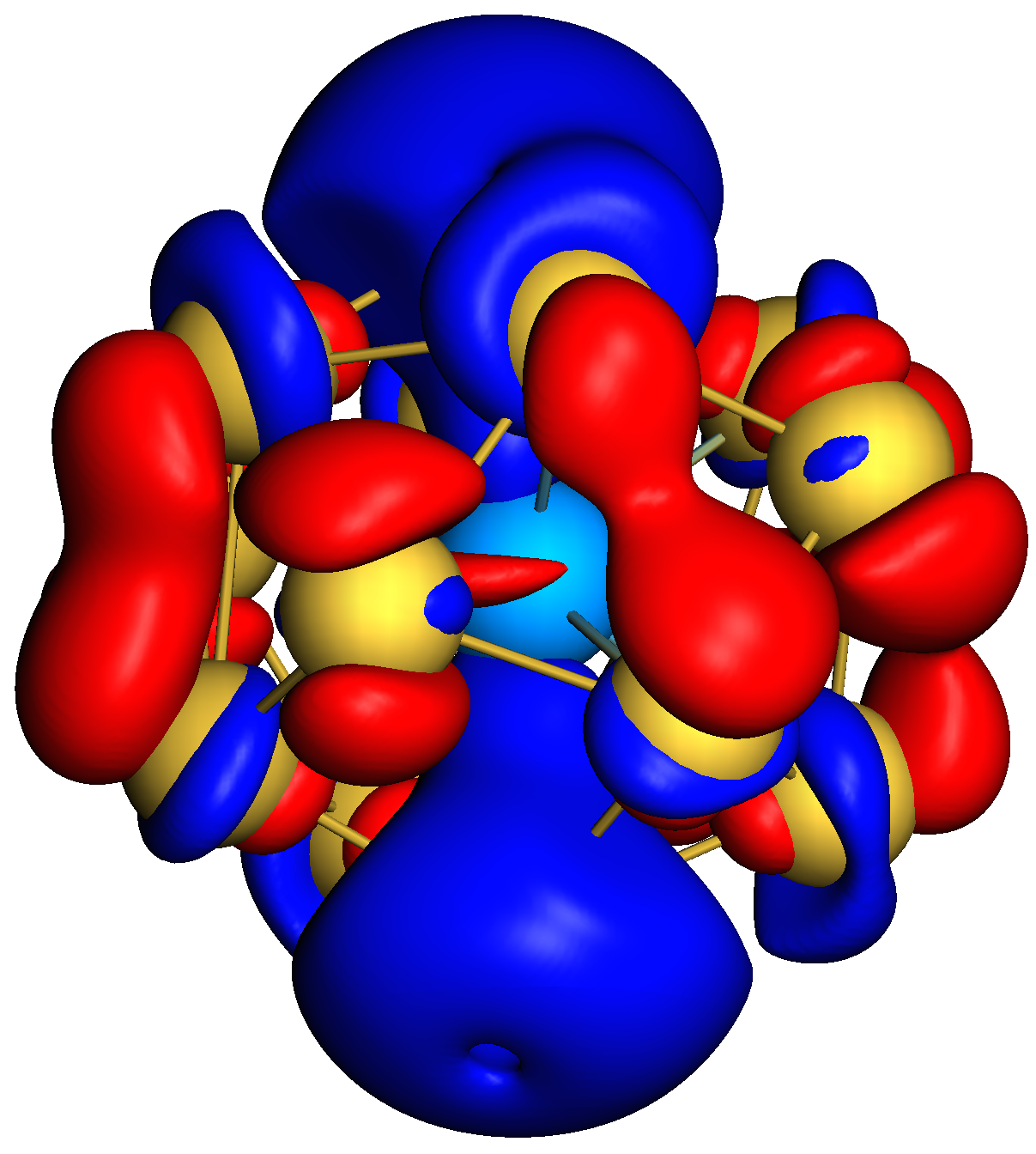**  HOMOα-1 | **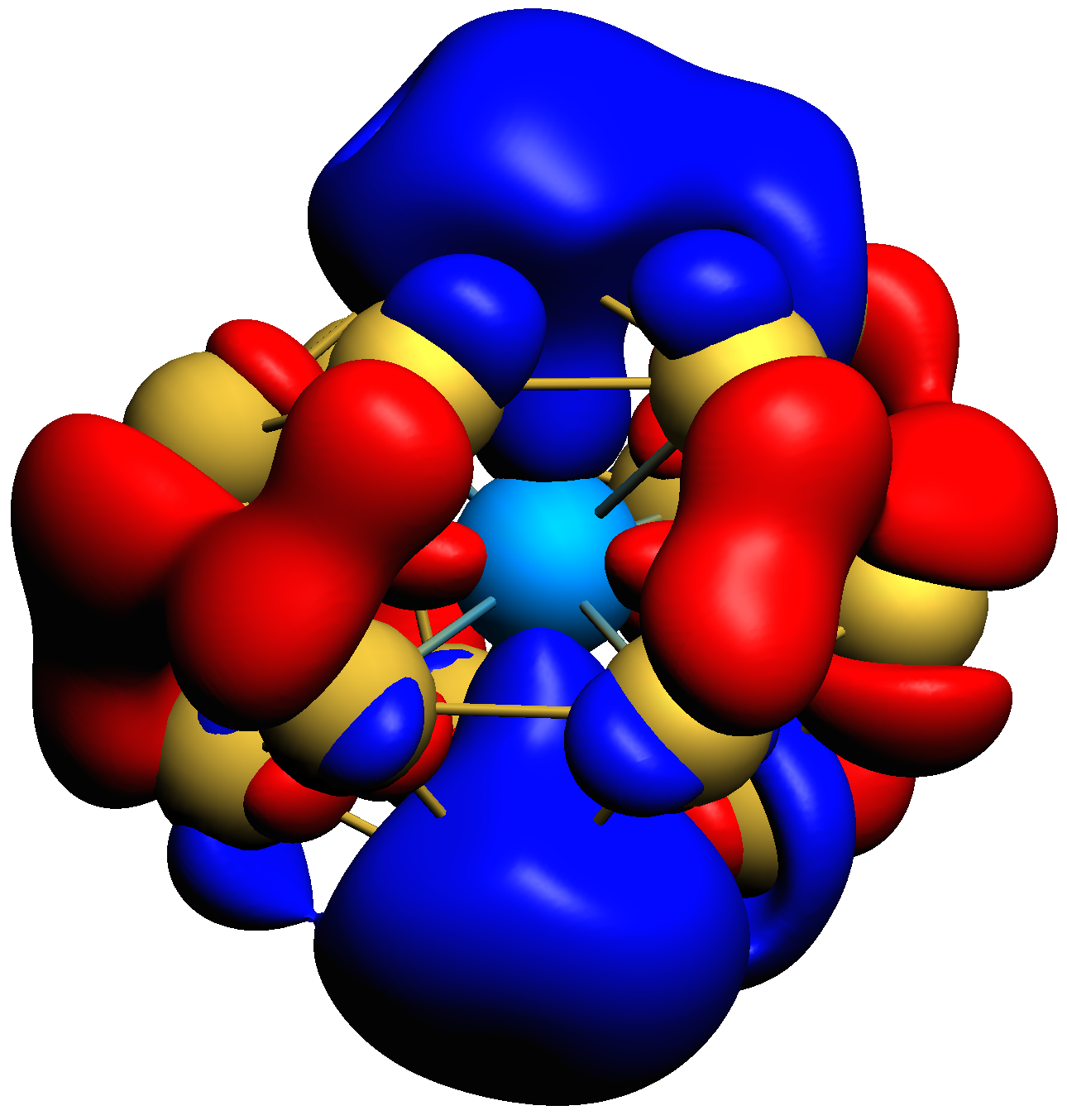**  HOMOα-2 | **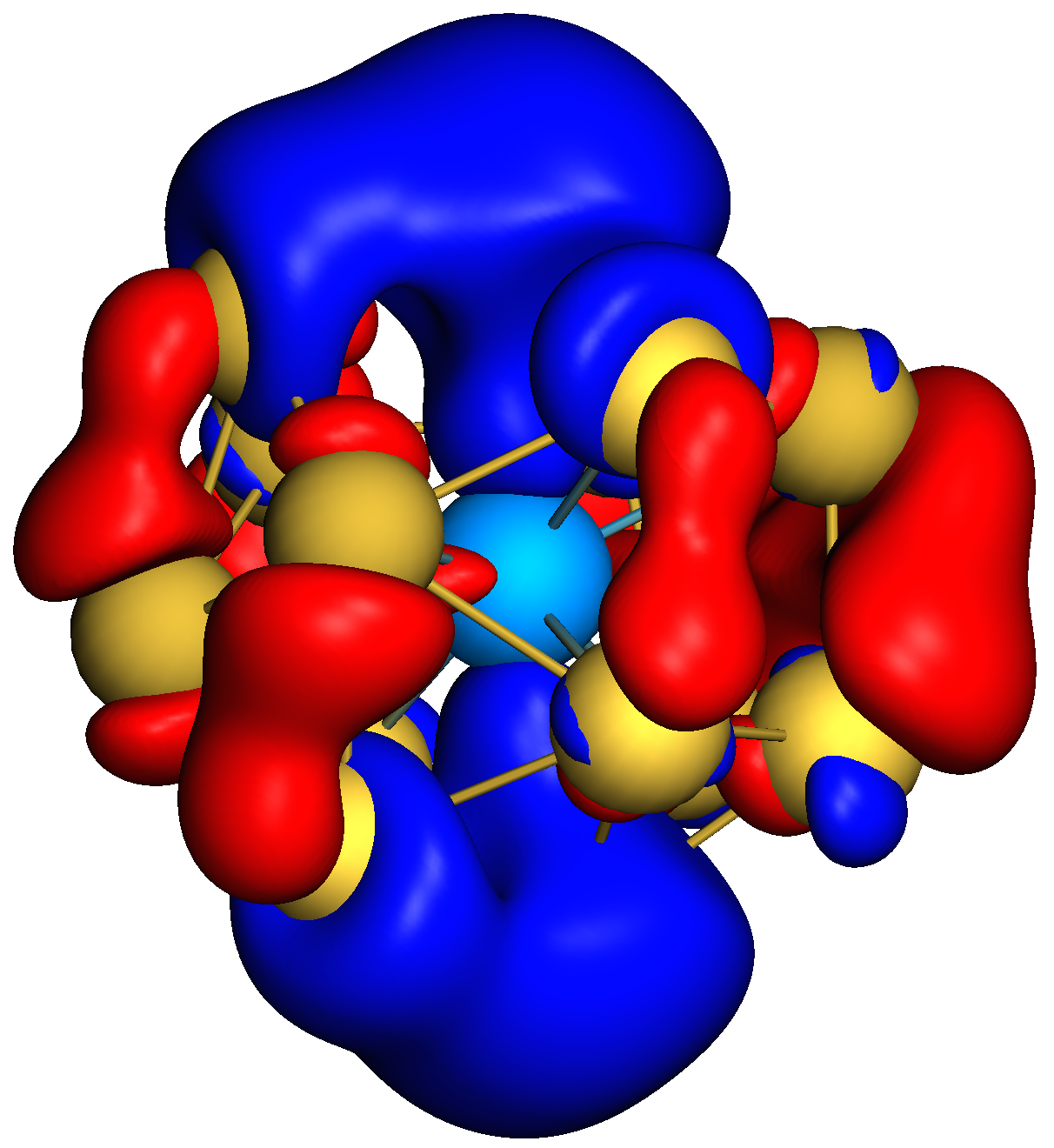**  HOMOα-4 |
| --- | --- | --- |
| **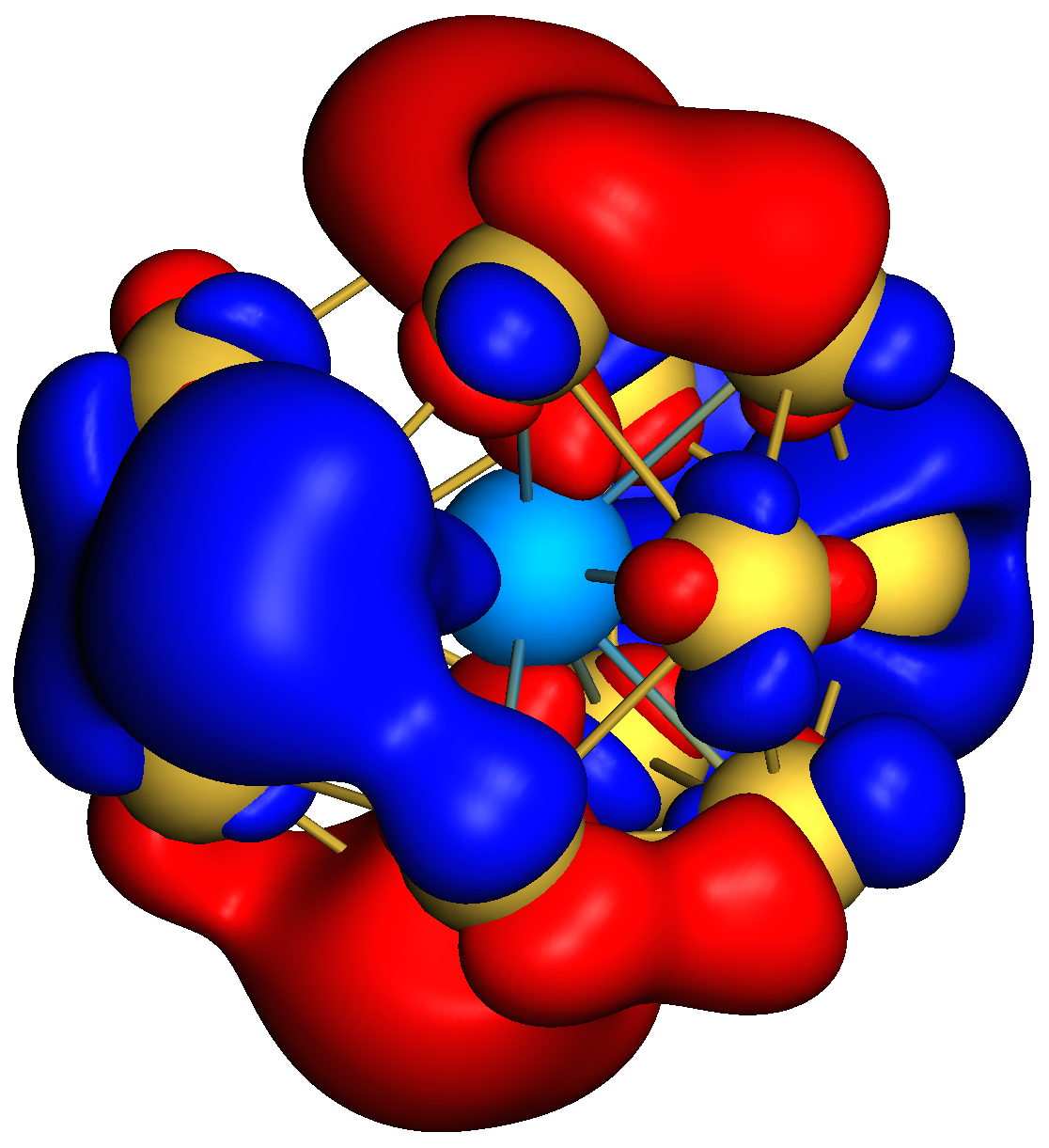**  HOMOα-5 | **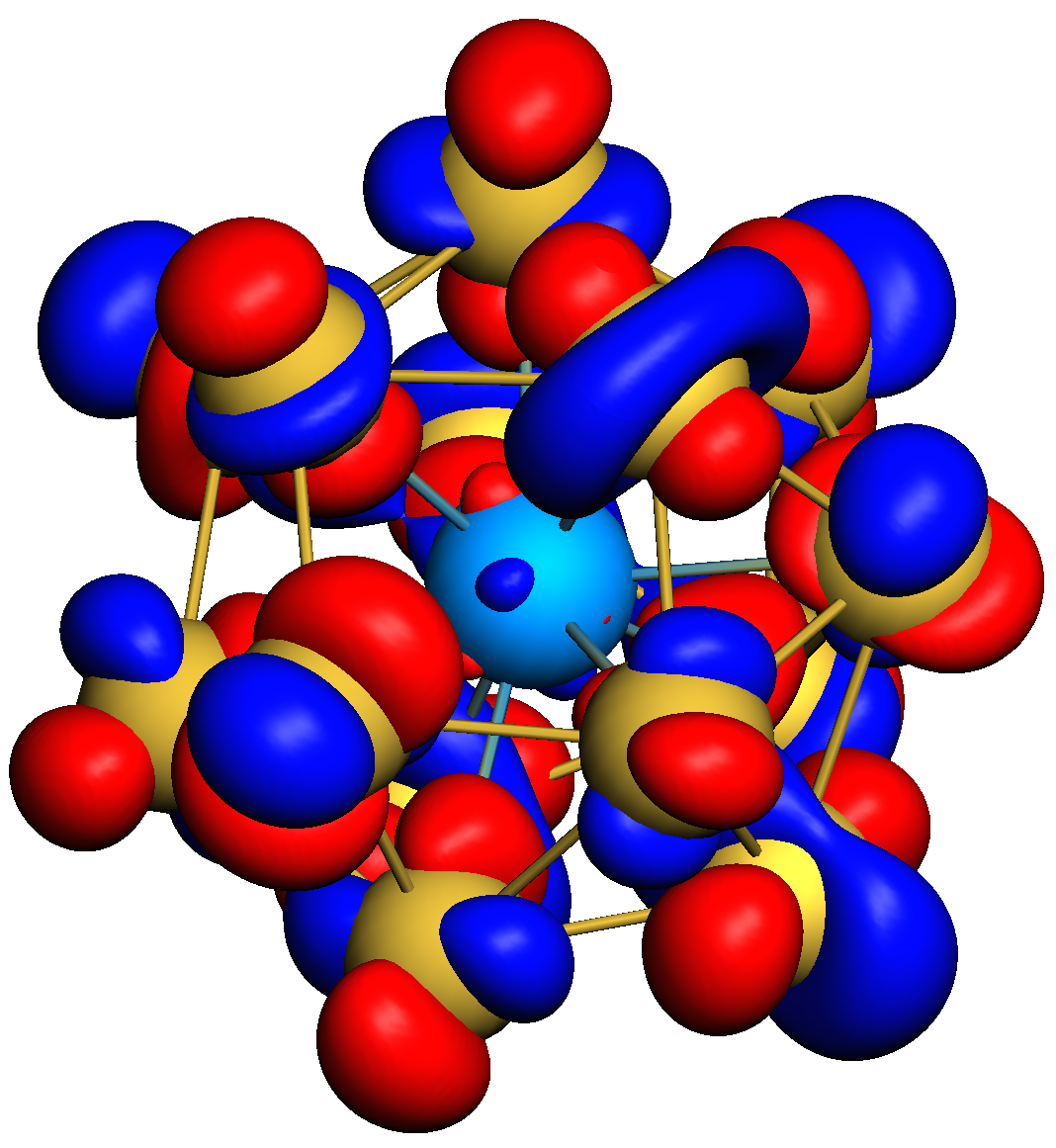**  HOMOα-30 | **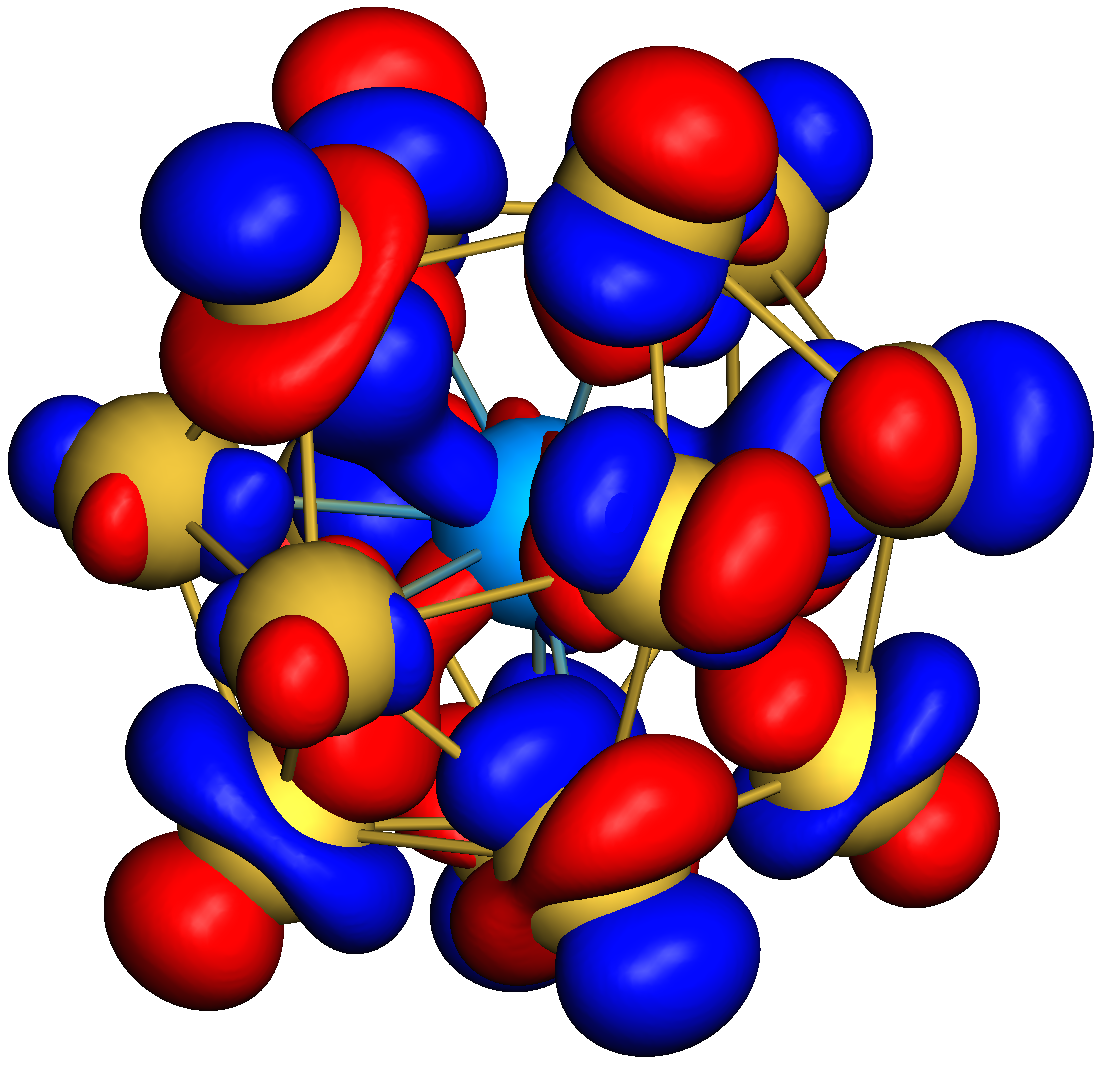**  HOMOα-34 |
| **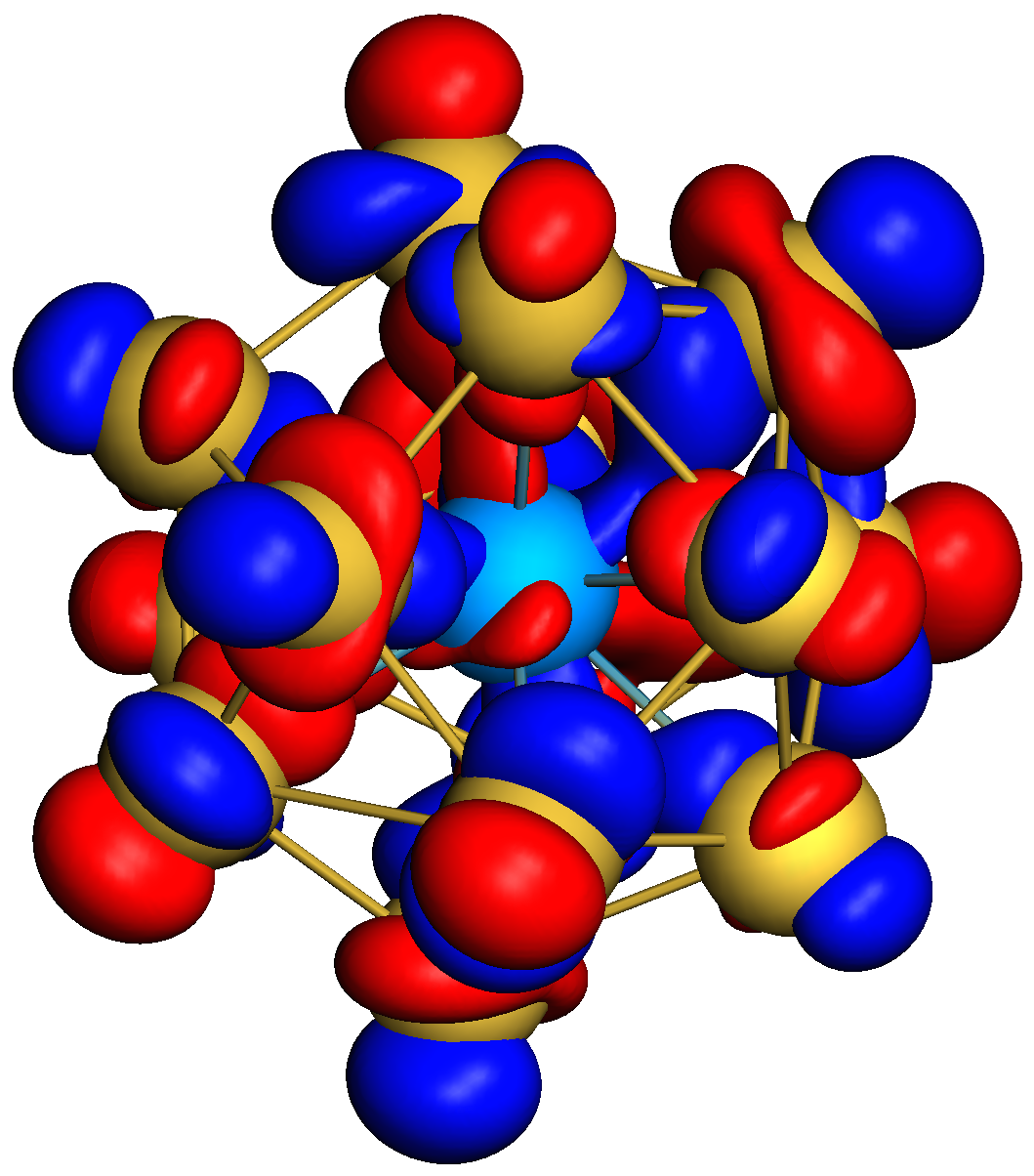**  HOMOα-35 | **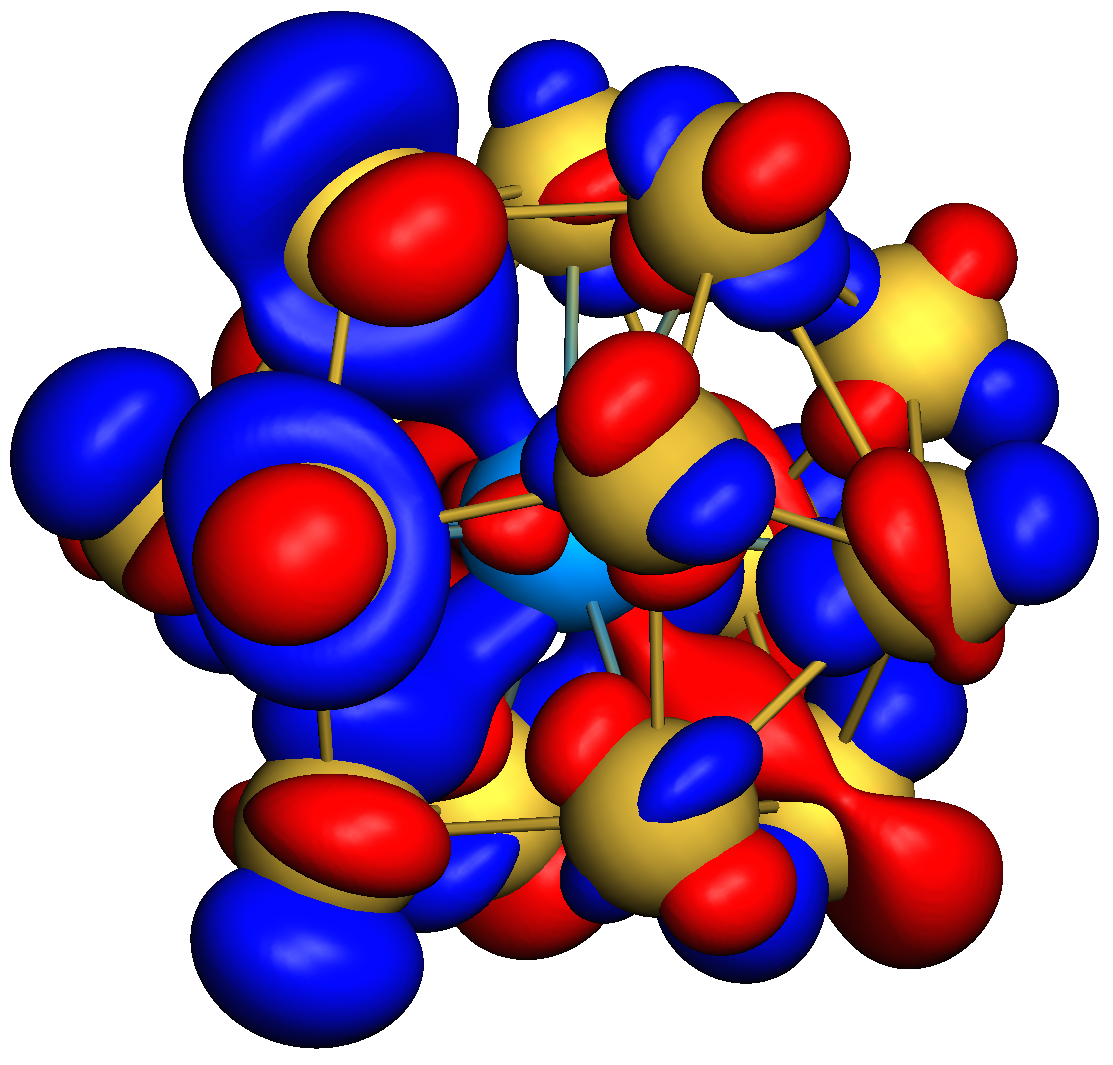**  HOMOα-37 | **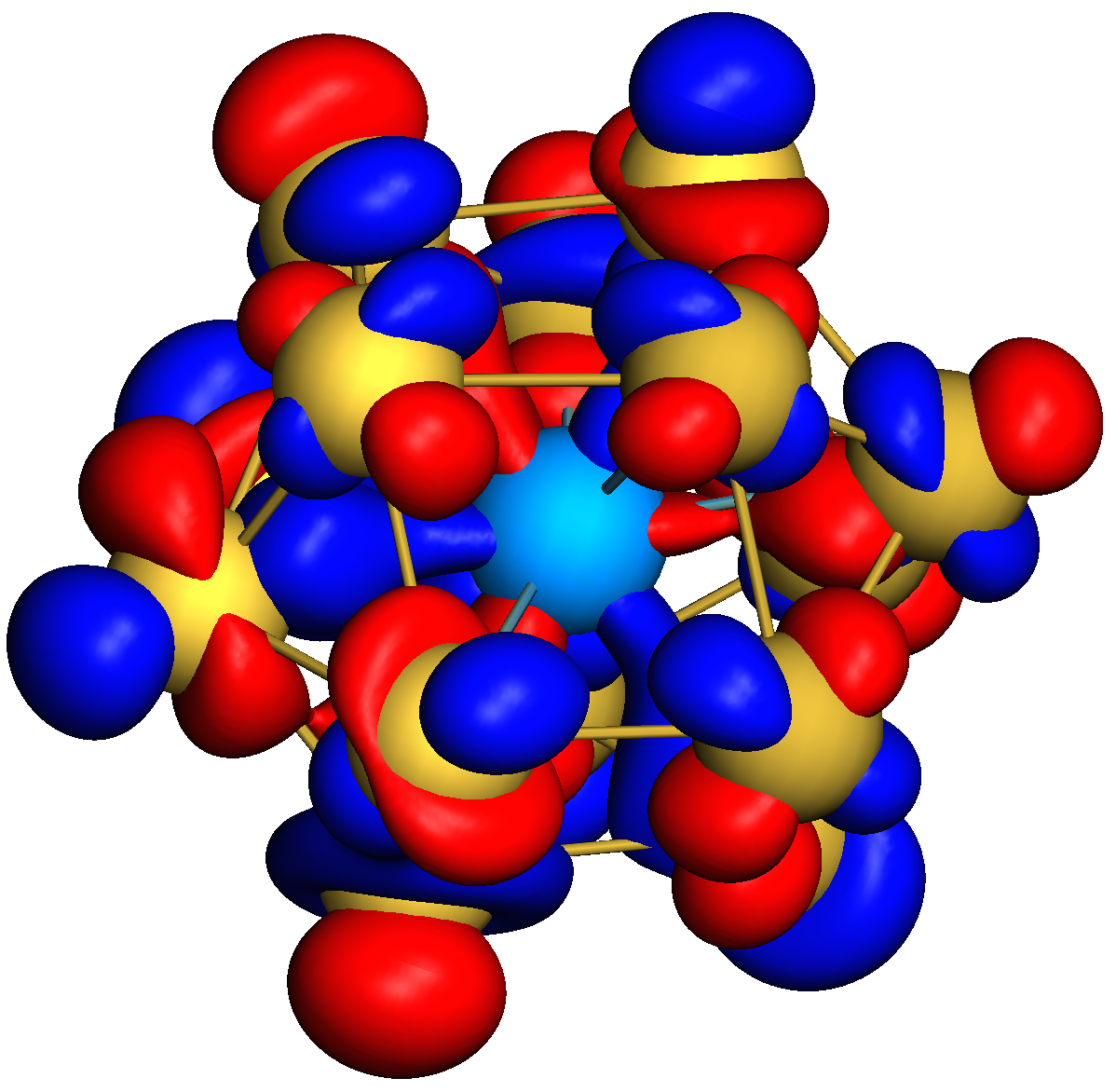**  HOMOα-39 |
| **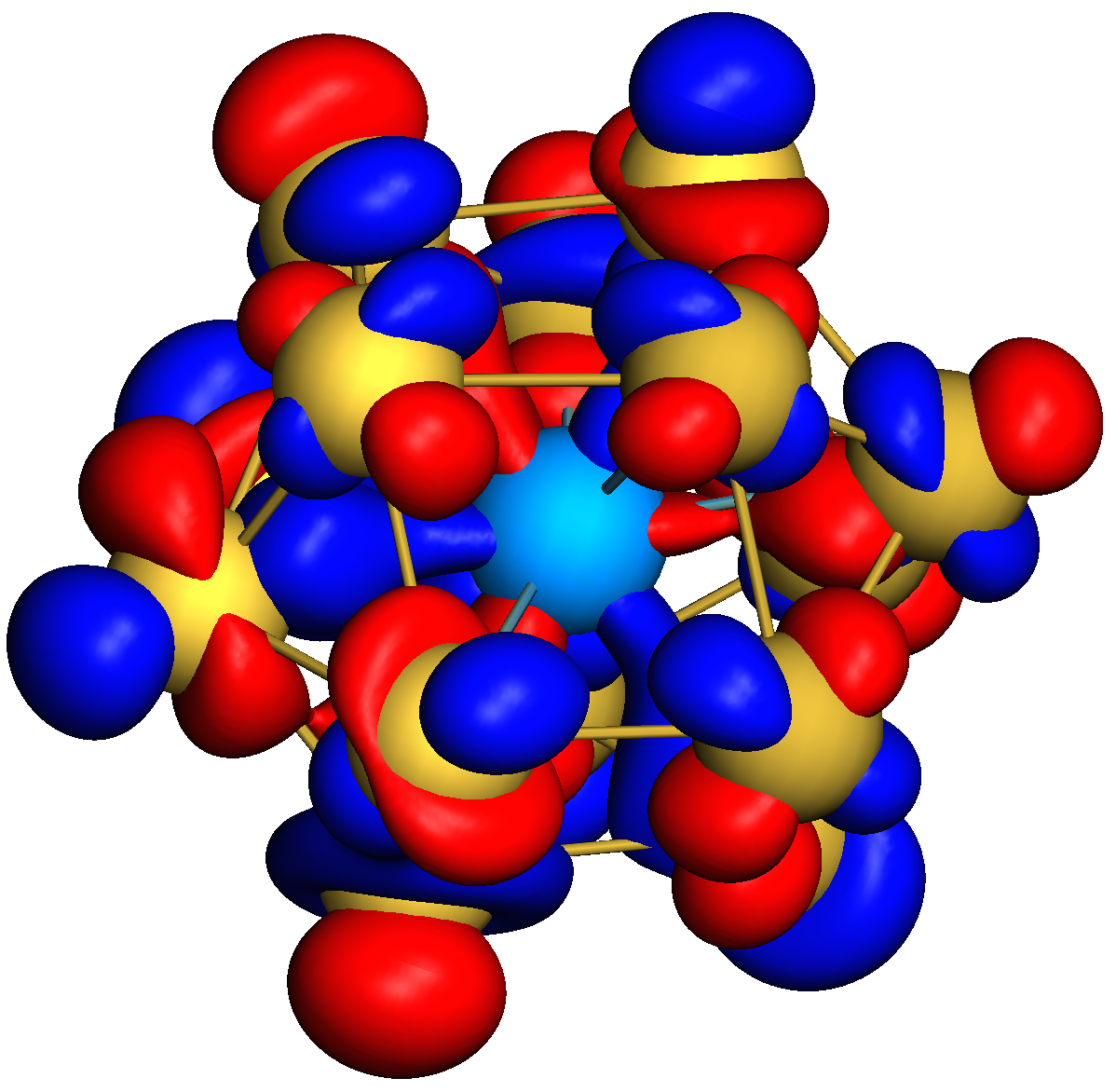**  HOMOα-41 | **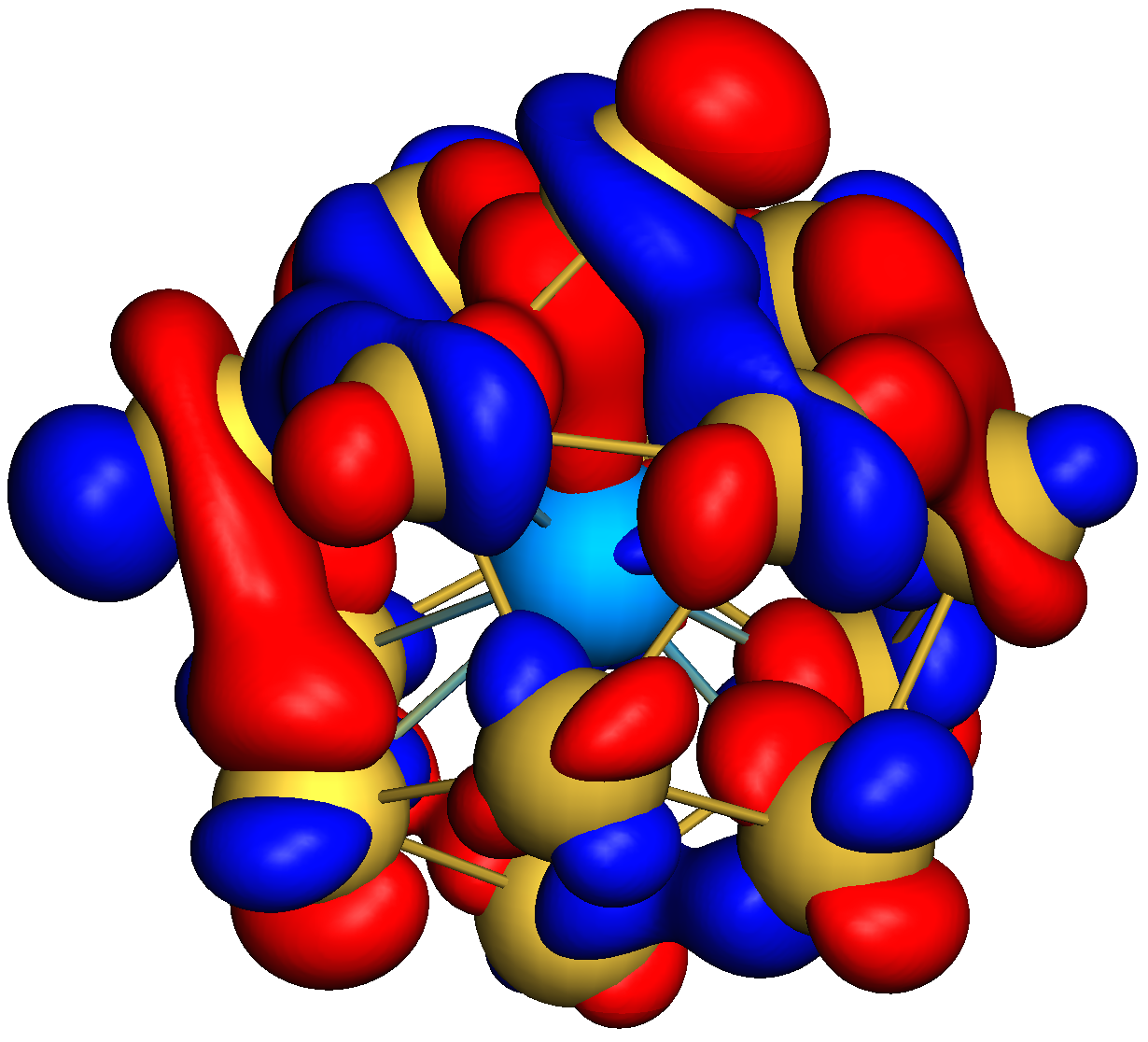**  HOMOα-45 | **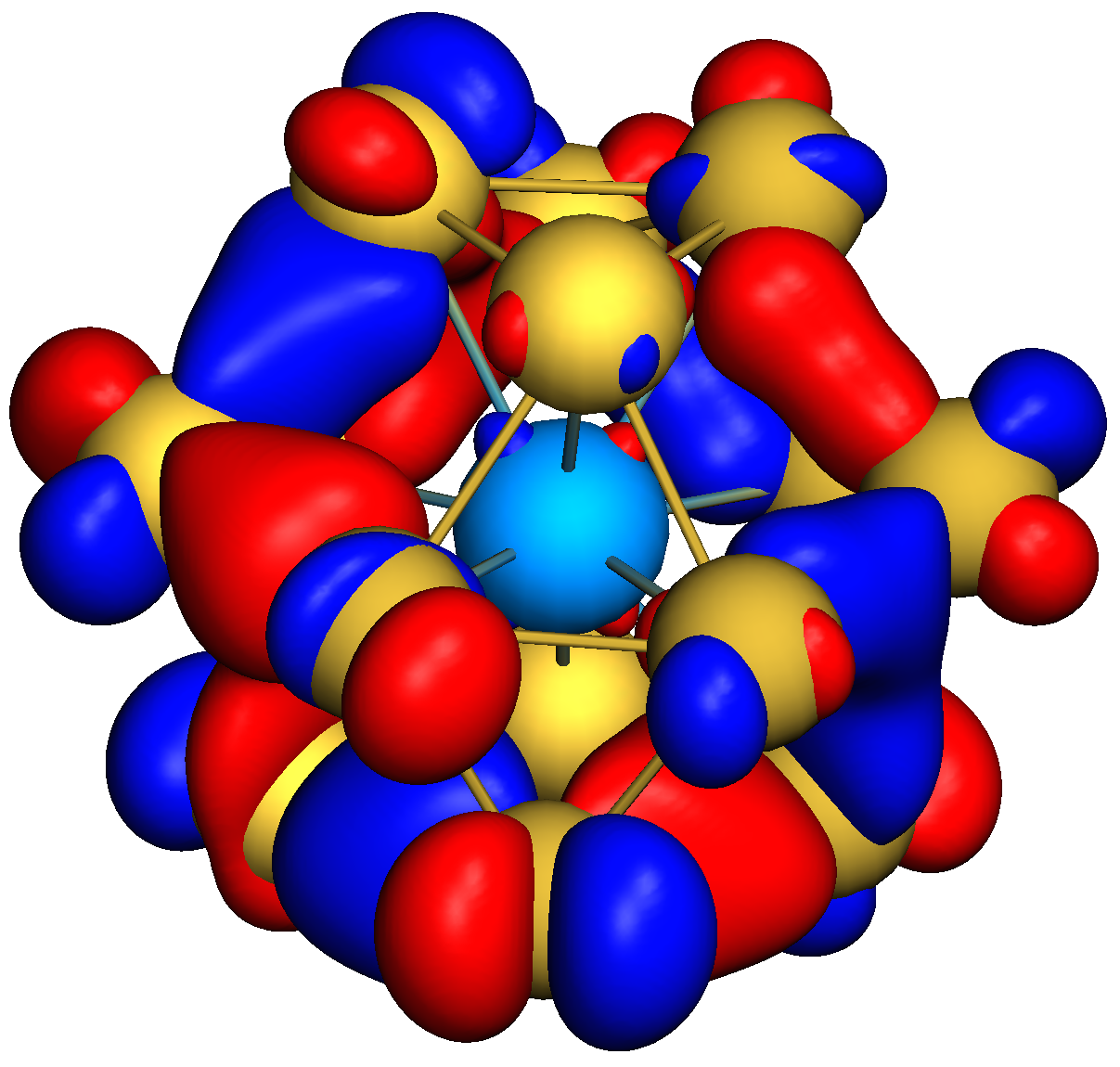**  HOMOα-58 |
| **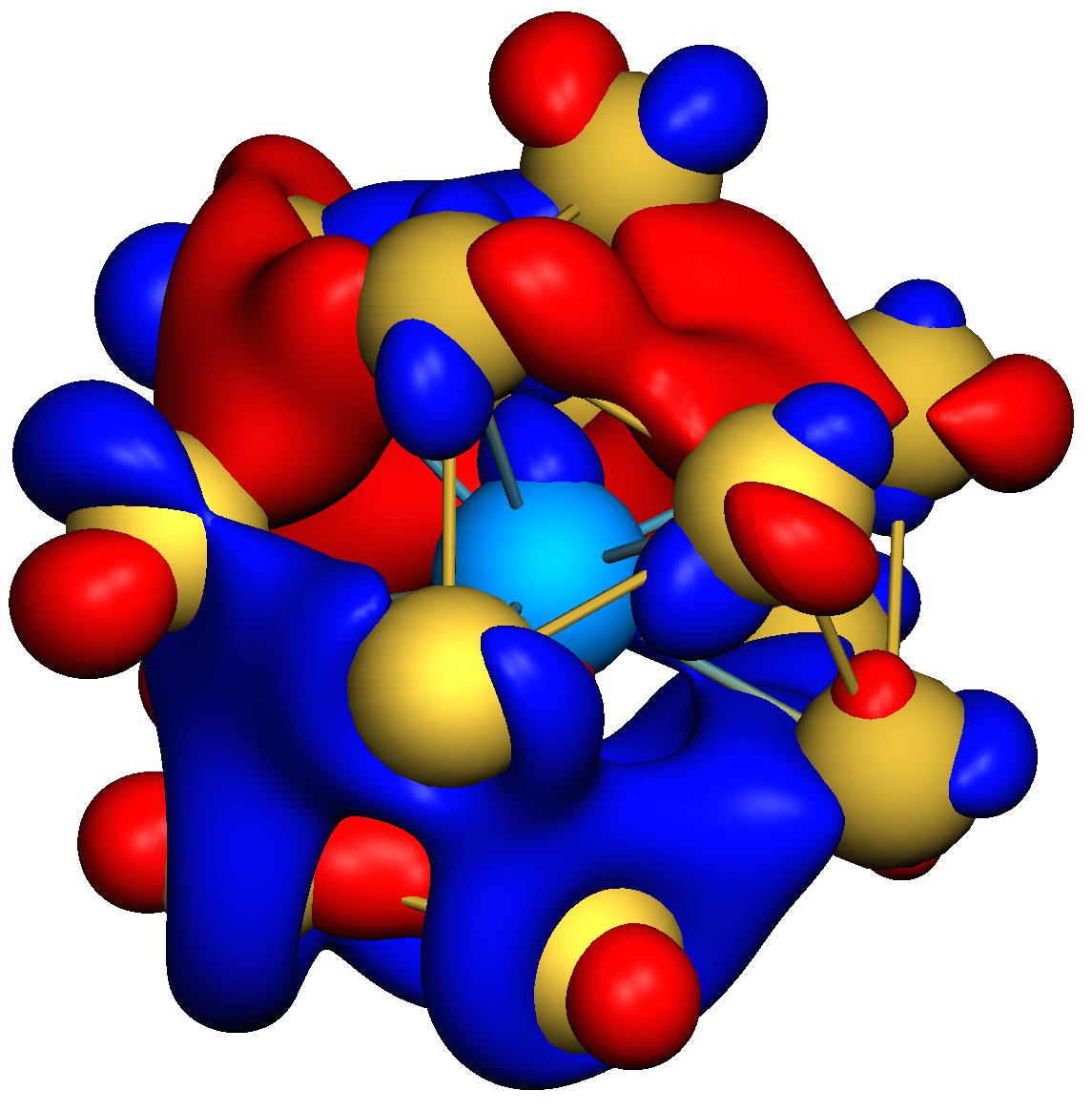**  HOMOα-66 | **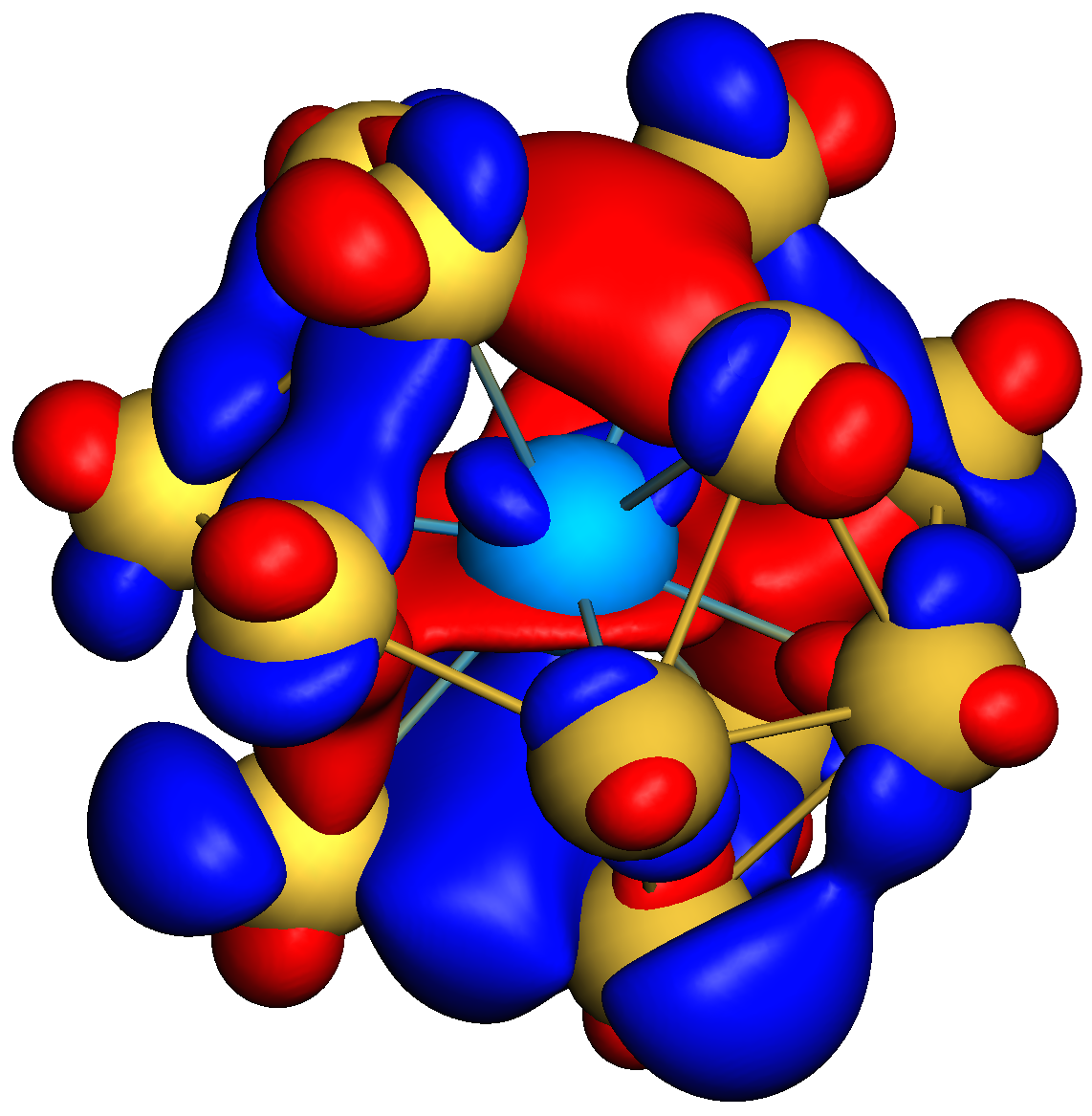**  HOMOα-67 | **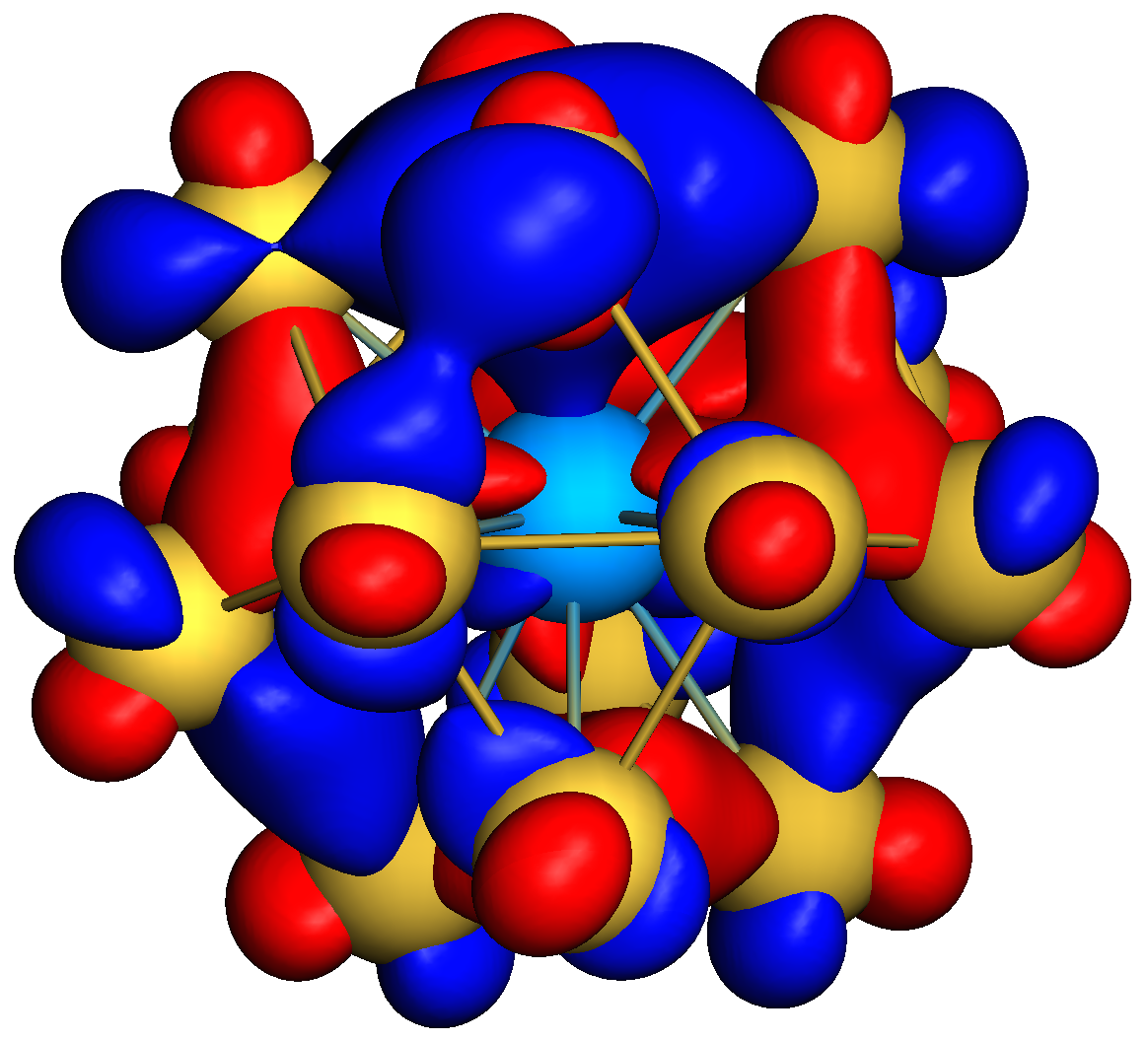**  HOMOα-68 |
| **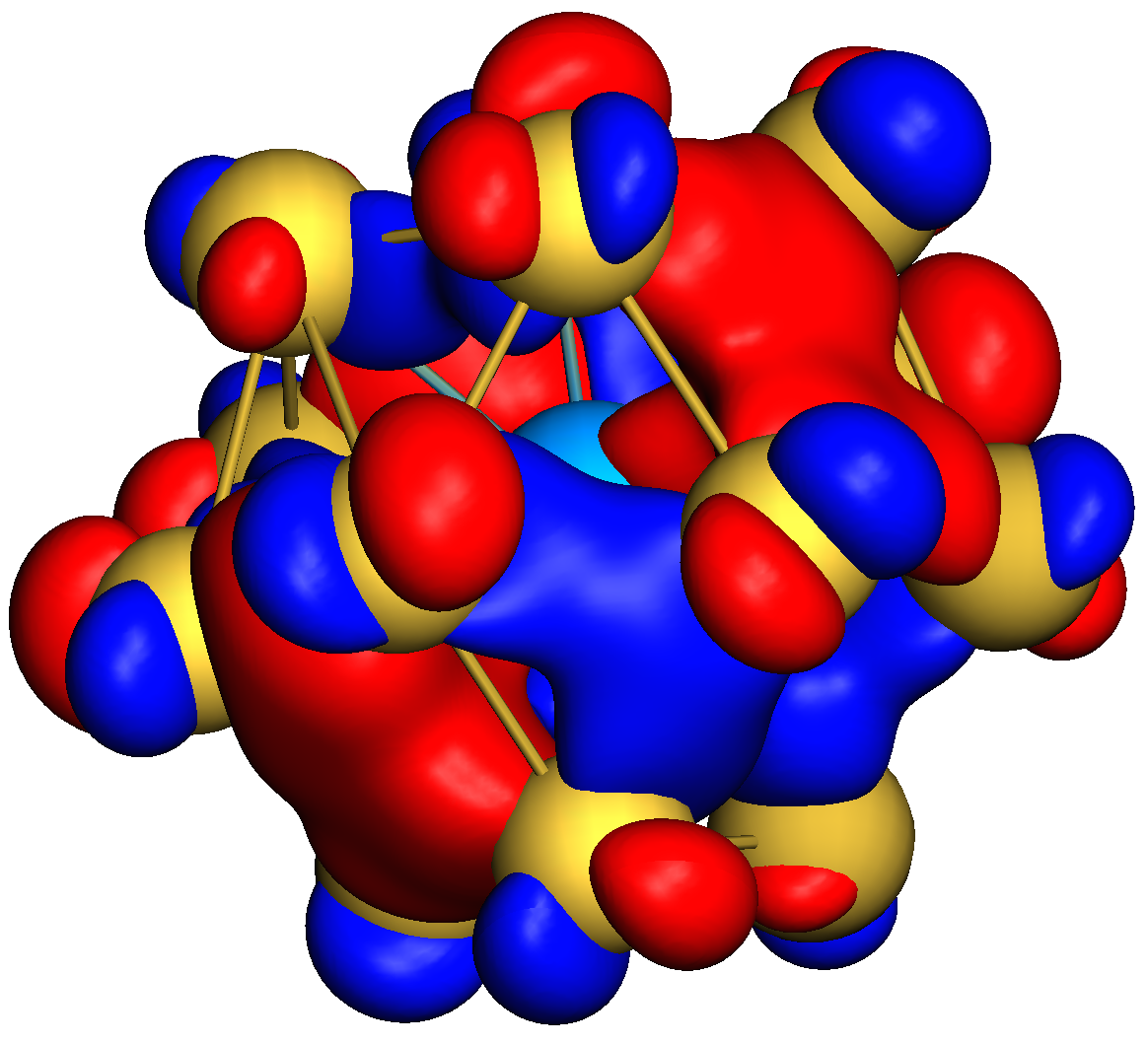**  HOMOα-69 | **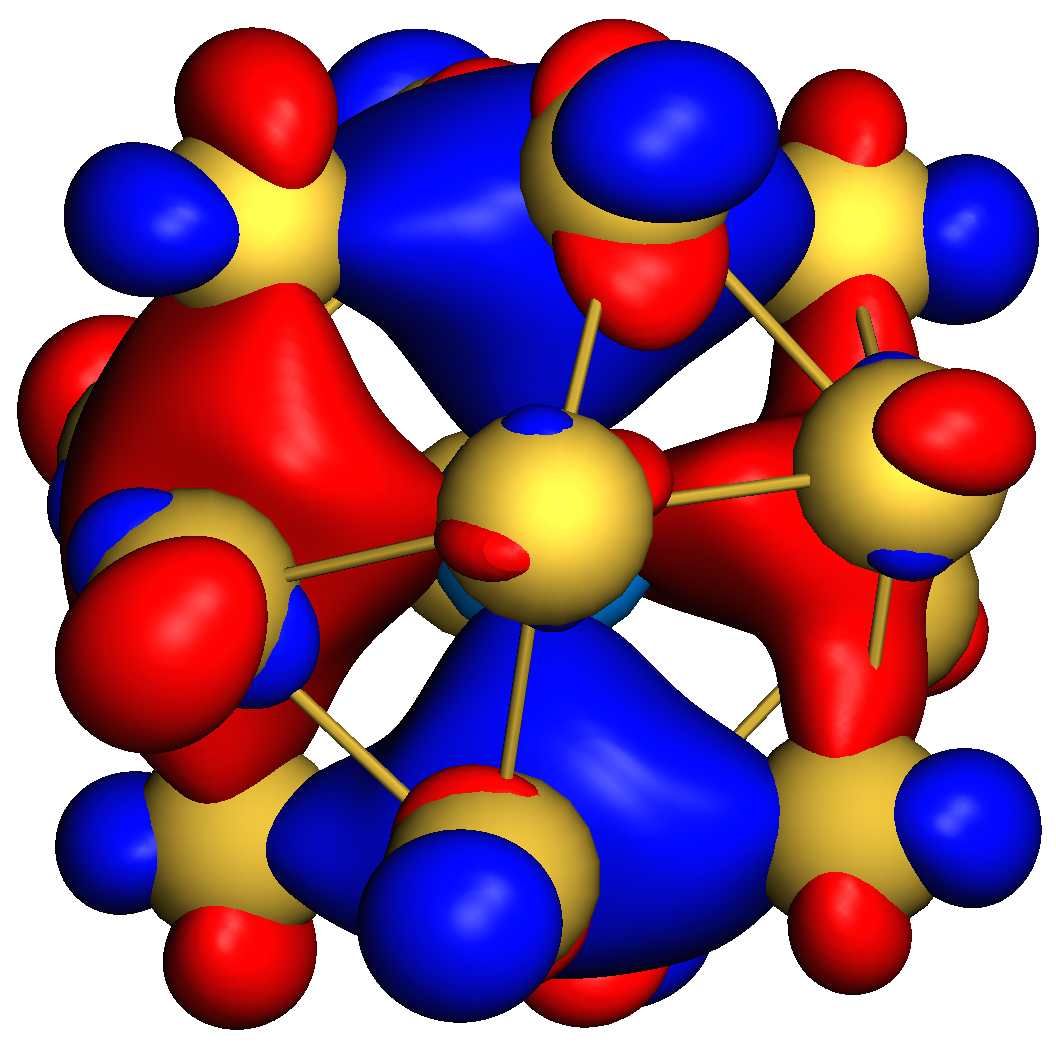**  HOMOα-74 | **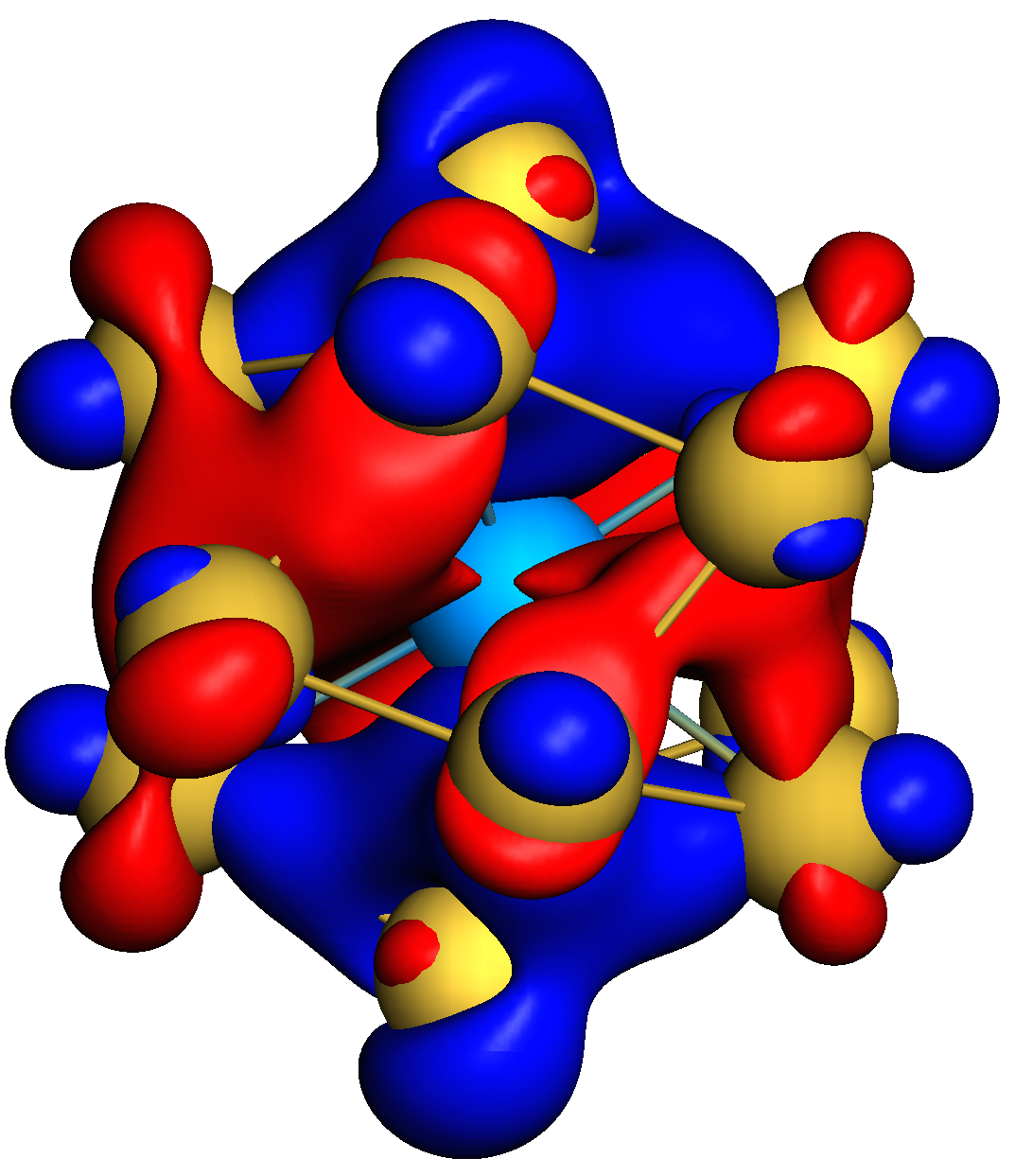**  HOMOα-75 |
| **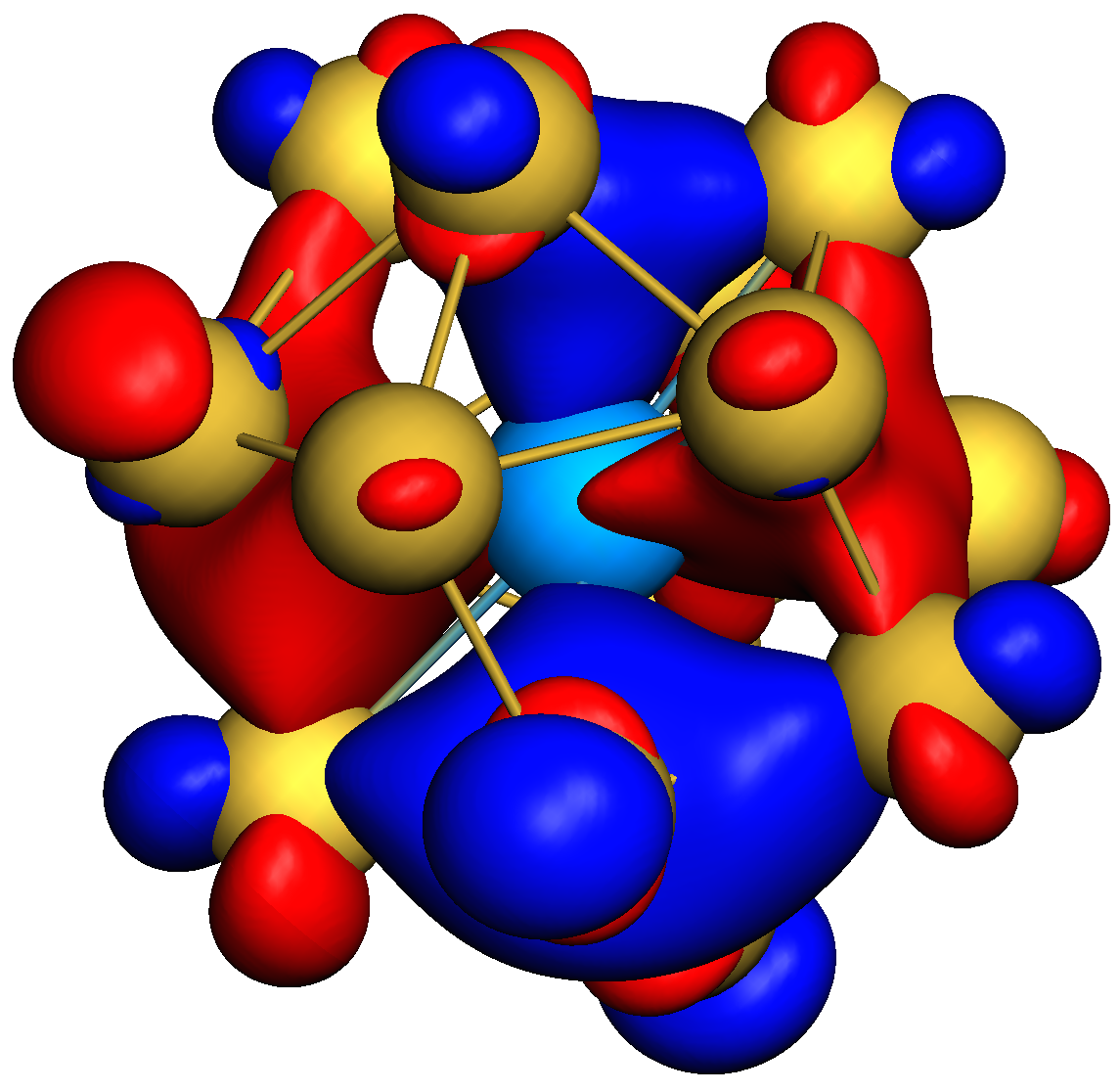**  HOMOα-76 | **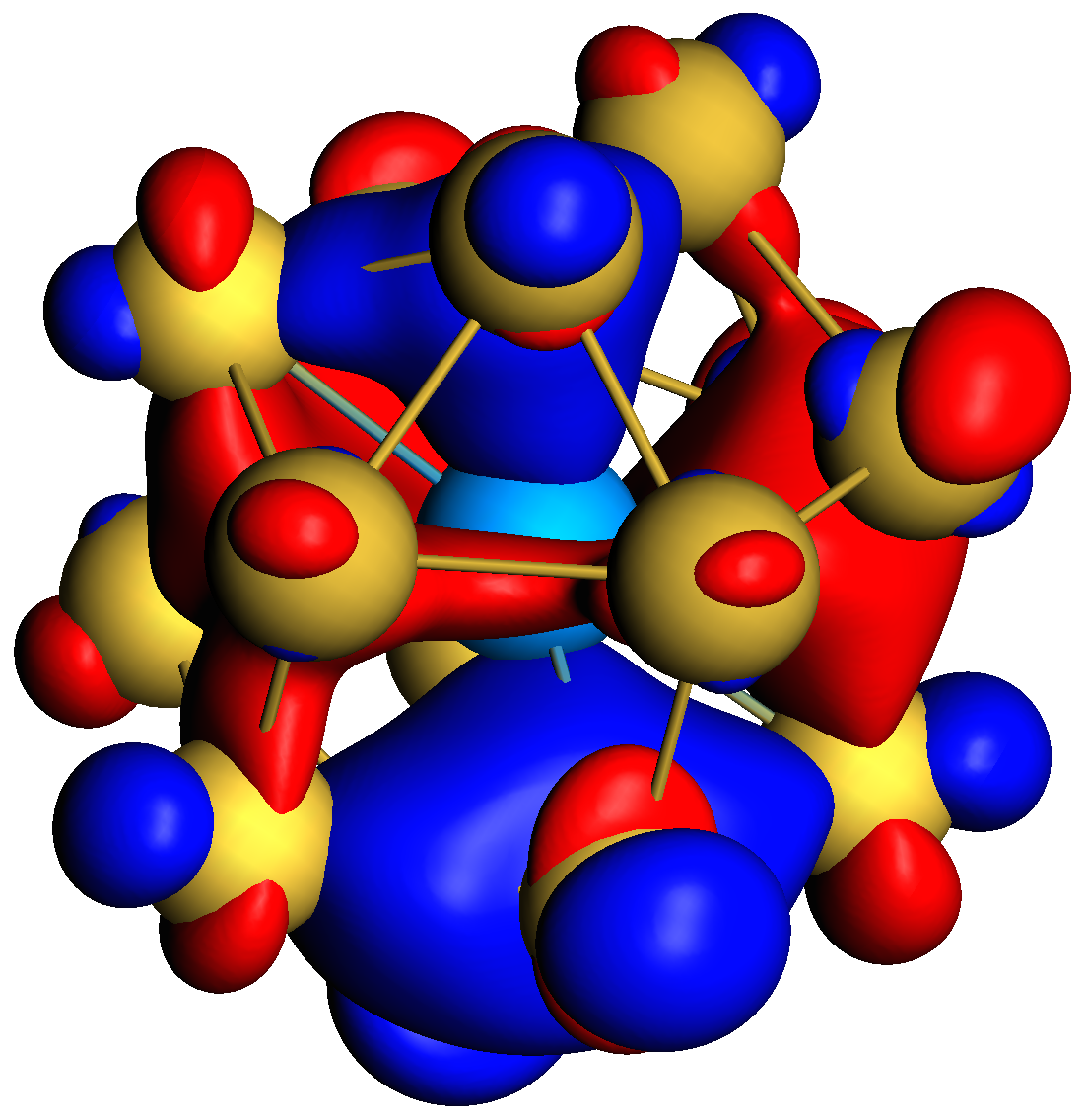**  HOMOα-77 | **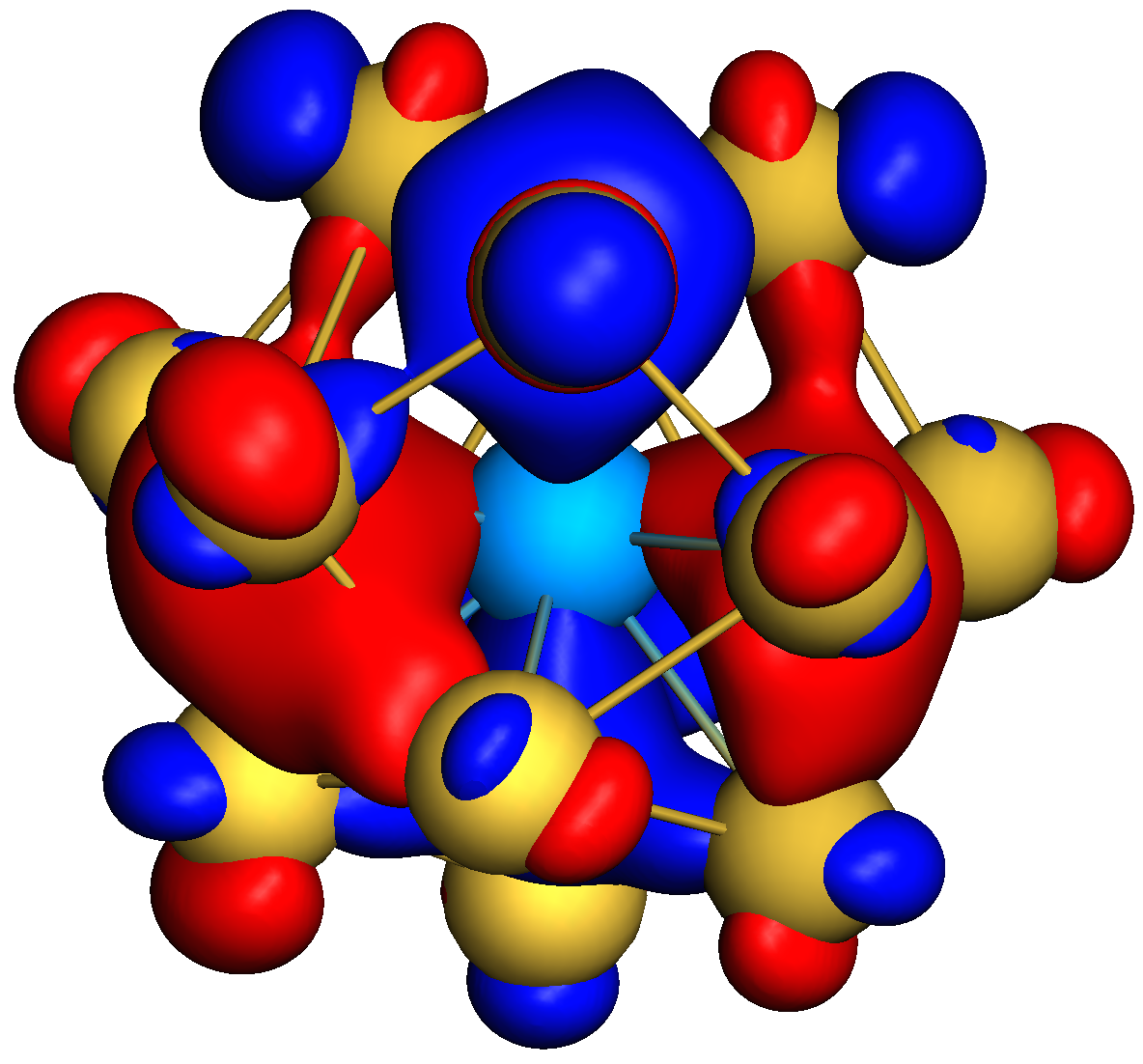**  HOMOα-78 |

**Reference:**

1. Zhang, M., Zhang, H., Zhao, L., Li, Y. & Luo, Y. Low-Energy Isomer Identification, Structural Evolution, and Magnetic Properties in Manganese-Doped Gold Clusters MnAu_n_ (n= 1–16). *J. Phys. Chem. C.* **116**, 1493-1502 (2012).
2. Hossain, D., Pittman, J. C. U. & Gwaltney, S. R. Structures and Stabilities of the Metal Doped Gold Nano-Clusters: M@ Au_10_ (M= W, Mo, Ru, Co). *J. Inorg. Organomet. Polym*. 2013, DOI:10.1007/s10904-013-9995-6.
